# Prevalence of anxiety and depression in young stroke patients, and associated factors: a meta-analysis

**DOI:** 10.3389/fneur.2026.1807072

**Published:** 2026-04-30

**Authors:** Yuxue Tong, Songmei Cao, Jingjing Wang, Yuan Qin, Jingxi Lin, Zhen Fang, Jing Qiu

**Affiliations:** Department of Nursing, Affiliated Hospital of Jiangsu University, Zhenjiang, Jiangsu, China

**Keywords:** stroke, young adults, anxiety, depression, associated factors, meta-analysis

## Abstract

**Objective:**

To systematically evaluate the prevalence of anxiety and depression among young stroke patients and their associated factors using a meta-analysis.

**Methods:**

In this review, young stroke was defined as stroke occurring in individuals aged 15–60 years. A comprehensive literature search was conducted in the China National Knowledge Infrastructure (CNKI), Wanfang Data, China Biomedical Literature Database, PubMed, Embase, Web of Science, the Cochrane Library, and Wiley for studies reporting the prevalence of anxiety and depression as well as their associated factors in young stroke patients. The search period spanned from database inception to September 2025. Meta-analysis was performed using RevMan 5.4, and publication bias analyses were conducted in Stata 17.0.

**Results:**

Twenty-six studies involving 5,634 patients were included, with 555 cases of anxiety and 1,334 cases of depression. Meta-analysis revealed that the prevalence rates of anxiety and depression among young stroke patients were 35% [95% CI (29–41%)] and 35% [95% CI (29–41%)], respectively. Subgroup analyses revealed the following: by publication year, 32% anxiety and 33% depression among young stroke patients from 2005 to 2018; 43% anxiety and 32% depression from January 2019 to September 2025. By country, the prevalence rates of anxiety and depression among young stroke patients in China were 35 and 34%, respectively, while those in other countries were 36 and 30%. By first-ever stroke, the prevalence rates among first-ever stroke patients were 32 and 31%, respectively, while those for non-first-ever patients were 37 and 34%. By gender, the prevalence rates of anxiety and depression among male patients were 32 and 36%, respectively, while those among female patients were 43 and 37%, respectively. Alcohol consumption and prior depressive symptoms showed relatively stable associations with anxiety in young stroke patients. Gender reached statistical significance in the primary analysis, but this finding was not robust in sensitivity analysis. National Institutes of Health Stroke Scale (NIHSS) score [OR = 3.22, 95% CI (2.04, 5.08)], alcohol consumption [OR = 3.15, 95% CI (1.85, 5.36)], lesion location [OR = 4.8, 95% CI (2.55, 9.06)], Herth Hope Index (HHI) score [OR = 1.96, 95% CI (1.42, 2.71)], Stroke-related shame (SSS) score [OR = 2.04, 95% CI (1.47, 2.81)], hypertension [OR = 1.64, 95% CI (1.31, 2.04)], diabetes [OR = 2.15, 95% CI (1.6, 2.88)], hyperlipidemia [OR = 1.53, 95% CI (1.2, 1.96)], monthly household income [OR = 1.93, 95% CI (1.18, 3.15)], lesion area [OR = 3.25, 95% CI (1.8, 5.87)], multiple lesions [OR = 2.31, 95% CI (1.51, 3.55)], and length of hospitalization [OR = 1.62, 95% CI (1.16, 2.27)] were identified as factors influencing depression in young stroke patients (*p* < 0.05).

**Conclusion:**

In conclusion, this review indicates that anxiety and depression are both common among young stroke patients and deserve greater attention in routine stroke care. Alcohol consumption appears to be a common factor associated with both anxiety and depression, while prior depressive symptoms may also be associated with anxiety. For depression, neurological severity, lesion-related characteristics, vascular comorbidities, and psychosocial factors were identified as potential associated factors. However, some findings, particularly those related to gender and several other exploratory variables, were not stable in sensitivity analyses and should therefore be interpreted with caution. More standardized, prospective, and longitudinal studies are needed to further clarify the mental health burden and associated factors in young stroke patients and to support earlier and more targeted psychological assessment and intervention in this population.

**Systematic review registration:**

https://www.crd.york.ac.uk/PROSPERO, identifier, CRD420251181939.

## 1 Introduction

Global Burden of Disease studies indicate that stroke remains one of the leading causes of death and long-term disability worldwide (1). Although stroke has traditionally been considered a disease of older adults, it is not uncommon in young adults, and in high-income countries, the burden of stroke in this population has not declined as consistently as that observed among older adults (2, 3). This issue is particularly important because young patients usually have a longer life expectancy after a first-ever stroke and therefore may face long-term consequences in terms of functional impairment, occupational disruption, family burden, and psychosocial stress (4).

Among the non-motor sequelae of stroke, anxiety and depression are among the most common and clinically significant neuropsychiatric complications (5). Previous systematic reviews in the general stroke population have reported that the prevalence of post-stroke anxiety within the first year is approximately 29.3% (6), and this proportion may be even higher during the very early stage after stroke onset. Likewise, a large systematic review and meta-analysis found that the overall prevalence of post-stroke depression was 27% (7), with estimates of 24% based on clinical interviews and 29% based on rating scales. More recent evidence further suggests that stroke survivors have nearly three times the risk of depression compared with the general population, and approximately one-third of patients experience post-stroke depression (8). Collectively, these findings underscore the clear clinical importance of routine psychological assessment in patients with stroke.

However, evidence derived from the general stroke population cannot be directly extrapolated to younger patients. At present, there is no universally accepted definition of “young stroke” in the literature; the upper age limit varies across studies, ranging from 15 to 24 years and < 55 years to as high as 60 years, and young-onset stroke is etiologically more heterogeneous than stroke occurring in older adults (9, 10). Against this background, the present study defined young stroke as 15–60 years for two main reasons. First, the available primary studies in this field themselves cover a relatively broad age range, and in some datasets, the upper age limit extends to 60 years. Second, this age range encompasses a broader segment of the working-age population—a stage of life in which a stroke can profoundly disrupt education, employment, child-rearing, social participation, and long-term economic productivity (11). In addition, young stroke survivors often experience stroke during critical stages of identity formation, career development, family responsibility, and social role functioning. These life-course characteristics may influence both the prevalence and associated factors of post-stroke anxiety and depression, and may also alter the clinical implications of psychological screening and intervention (12).

To date, most systematic reviews have focused on the overall stroke population without age restriction, or have predominantly involved older patients, whereas evidence specifically addressing young stroke patients remains fragmented (6). Therefore, the present meta-analysis systematically searched the relevant literature from China and abroad to estimate the prevalence of anxiety and depression among young stroke patients and to synthesize their associated influencing factors, with the aim of providing evidence to support more targeted psychological care strategies and early intervention in clinical practice.

## 2 Methods

### 2.1 Literature search

The search strategy followed the PRISMA 2020 statement, and the completed PRISMA 2020 checklist is provided in the Supplementary Materials. Computerized searches were conducted in the China National Knowledge Infrastructure (CNKI), Wanfang Data databases, China Biomedical Literature Database, PubMed, Embase, Web of Science, Cochrane Library, and Wiley for published literature on the prevalence of anxiety and depression among young stroke patients and their associated factors. The search period spanned from database inception to September 2025. A combination of subject headings and free-text terms was employed. The search strategy included both the Chinese and the English languages. Because some database indexing systems classify late adolescents and young adults under overlapping subject terms, broad terms such as “Adolescent,” “youth,” and “young” were included at the retrieval stage to maximize sensitivity; however, studies involving participants aged < 15 years were excluded during full-text screening according to the predefined eligibility criteria.

The search terms were as follows: (Young Adult OR Adolescent OR youth OR young) AND (Strokes OR Cerebrovascular Apoplexy OR Apoplexy, Cerebrovascular OR Cerebrovascular Stroke OR Cerebrovascular Strokes OR Stroke, Cerebrovascular OR Strokes, Cerebrovascular OR Apoplexy OR Cerebral Stroke OR Cerebral Strokes OR Stroke, Cerebral OR Strokes, Cerebral) AND (anxiety OR depression OR negative emotions OR depressive).

### 2.2 Inclusion and exclusion criteria

The inclusion criteria were as follows: (1) Participants: stroke patients with study-defined young or young/middle-aged onset, with eligible age ranges broadly centered on 15–60 years; studies were retained when the reported sample fell largely within this range or when the original article explicitly defined the cohort as young or young/middle-aged stroke; (2) Study design: Observational studies, including cohort studies, case–control studies, and cross-sectional studies; (3) Outcomes: Prevalence and/or associated factors of anxiety and depression in young stroke patients. (4)Language: Chinese or English.

The exclusion criteria included the following: (1) Literature inconsistent with the study scope; (2) Full-text unavailable; (3) Conference abstracts, reviews, case reports, etc.; (4) Duplicate publications; (5) Low-quality literature.

### 2.3 Literature screening and data extraction

Two trained researchers independently screened and extracted data from the literature, strictly adhering to inclusion and exclusion criteria. Retrieval results were imported into EndNote software, where duplicate records were removed. Initial screening involved reviewing titles and abstracts, followed by full-text review to determine final inclusion. Disagreements were resolved through consultation with a third researcher. Data extraction encompassed the following: first author, publication year, country, study design, sample size, age, gender, number of participants experiencing anxiety/depression, assessment tools, and factors influencing anxiety and depression.

### 2.4 Literature quality assessment

Two researchers independently assessed literature quality. Cross-sectional studies were evaluated using the Agency for Healthcare Research and Quality (AHRQ) recommended risk of bias assessment criteria, with a total score of 11 points: 8–11 points indicated high-quality literature, 4–7 points moderate-quality literature, and 0–3 points low-quality literature (13). Cohort studies/case–control studies were assessed using the Newcastle-Ottawa Scale (NOS), with a total score of 9 points. Scores of 7–9 indicated high-quality studies, 4–6 indicated moderate-quality studies, and 0–3 indicated low-quality studies (14). Disagreements during evaluation were resolved through consultation with a third researcher.

### 2.5 Statistical methods

RevMan 5.4 software was employed for statistical analysis of included literature data. Odds ratios (ORs) with 95% confidence intervals (CIs) were used as the effect measures for associated factors, and pooled prevalence with 95% CIs was calculated for single-arm prevalence outcomes. Heterogeneity among studies was assessed using the Q test and the I^2^ statistic. When *p* > = 0.10 and I^2^ < 50%, a fixed-effects model was used; otherwise, a random-effects model was applied. Sensitivity analysis was conducted to explore sources of heterogeneity. Publication bias was assessed using funnel plots in RevMan 5.4 and Egger’s test (and trim-and-fill analysis, when applicable) in Stata 17.0. A *p*-value < 0.05 was considered statistically significant.

## 3 Results

### 3.1 Literature screening outcomes

**Figure 1:**
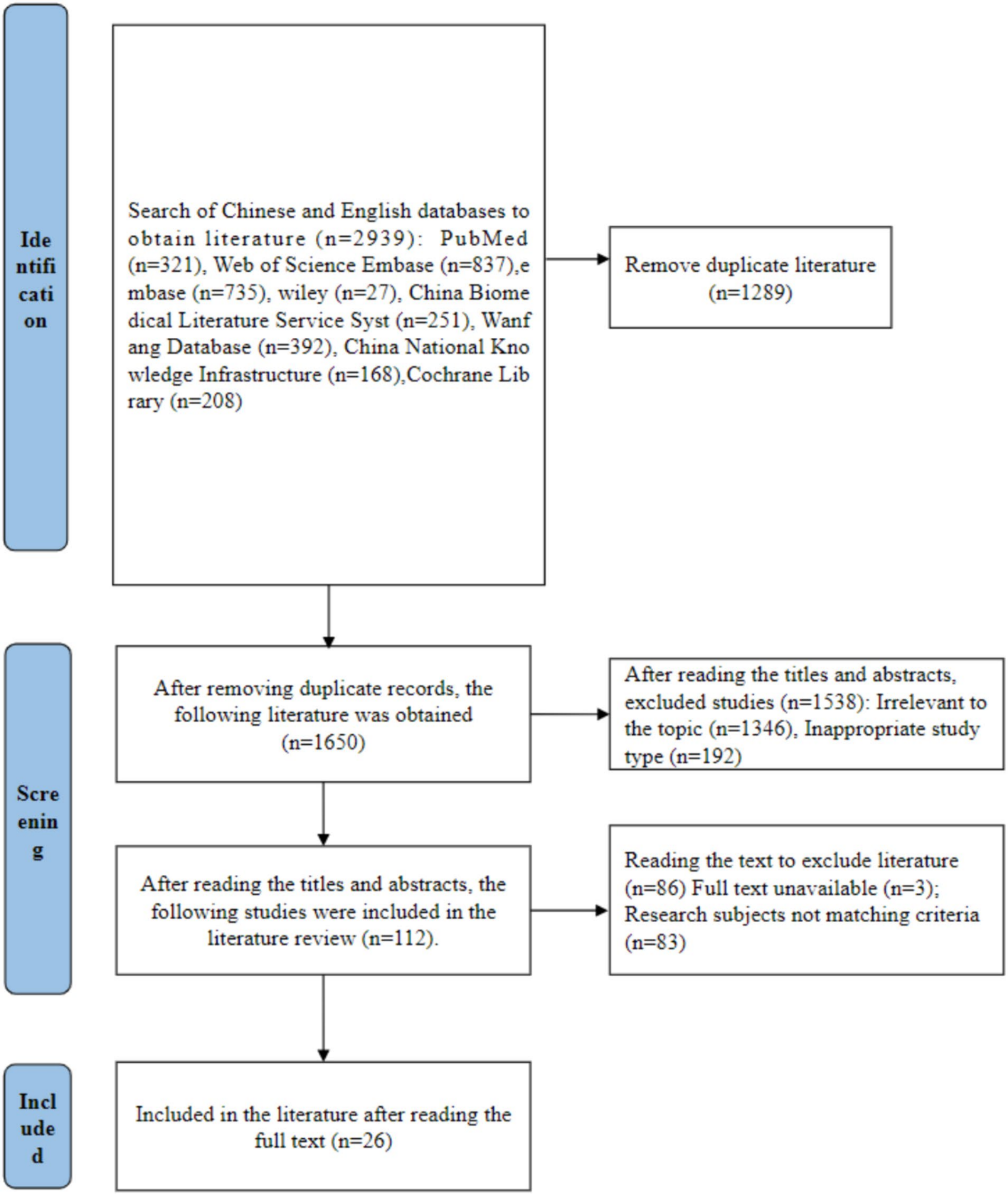
Literature screening process.

Initial searches yielded 2,939 relevant publications. After excluding 1,289 duplicates and reviewing titles, abstracts, and full texts, 26 studies (9, 10, 15–38) were ultimately included: 17 Chinese-language and 9 English-language articles. These studies involved 5,634 patients, including 555 with anxiety and 1,334 with depression. To avoid ambiguity, duplicate cohorts extracted from the same publication were labeled separately as independent data entries when the original article reported distinct groups or assessment points (e.g., Li 2014 1/2014 2 and Modino 2025 1/2025 2). The literature screening process is illustrated in Figure 1, and the characteristics and quality assessment of the included studies are presented in Tables 1, 2.

**Table 1:** Literature characteristics and quality evaluation of anxiety in young stroke patients.

First author	Year	Country	Study type	Sample size (cases)	Age (years)	Gender (male/female)	Anxiety [n(%)]	Assessment tool	Influencing factors	Quality (score)
Zhou et al. (15)	2010	China	Cross-sectional	61	18–59	-	16(0.2623)	HAMA	①②④	6
Li et al. (9)	2014	China	Cross-sectional	107	28–60	69/38	45(0.4206)	SAS	⑤⑦⑥⑧	8
Li et al. (9)	2014	China	Cross-sectional	107	28–60	69/38	28(0.2617)	SAS	⑤⑦⑥⑧	8
Wang et al. (16)	2014	China	Cross-sectional	69	18–45	56/13	25(0.3623)	HAMA	①⑦⑧	5
Koivunen et al. (17)	2015	Netherlands	Cross-sectional	130	16–49	67/73	52(0.4000)	HADS	⑨⑩⑪	7
Song et al. (18)	2015	China	Cross-sectional	78	28–45	43/35	33(0.4231)	SAS	⑧	6
Maaijweea et al. (10)	2016	Netherlands	Cross-sectional	511	18–50	224/287	118(0.2309)	HADS	②④⑥⑫	9
Ding (19)	2018	China	Cross-sectional	205	18–55	167/38	47(0.2293)	SAS	⑫	6
Zhang et al. (20)	2019	China	Cross-sectional	248	40–60	168/80	117(0.4718)	SAS	②③	9
Hannah et al. (21)	2022	Philippines	Cross-sectional	114	18–49	66/48	39(0.3421)	HADS	⑨⑫	6
Modino et al. (22)	2025	Spain	Cross-sectional	40	18–50	-	18(0.4500)	HAMA	⑦⑨⑬	8
Modino et al. (22)	2025	Spain	Cross-sectional	41	18–59	24/17	17(0.4146)	HAMA	⑦⑨⑬	8

HAMA, Hamilton Anxiety Rating Scale; HADS, Hospital Anxiety and Depression Scale; SAS, Self-Rating Anxiety Scale; ① NIHSS score, ② age, ③ gender, ④ educational attainment, ⑤ smoking status, ⑥ alcohol consumption, ⑦ presence of comorbidities, ⑧ lesion location, ⑨ degree of functional impairment, ⑩ pain-related indicators, ⑪ surgical intervention, ⑫ prior depressive symptoms, ⑬ occupational background.

**Table 2:** Literature characteristics and quality evaluation of depression in young stroke patients.

First author	Year	Country	Study type	Sample size (cases)	Age (years)	Gender (male/female)	Depression [n(%)]	Assessment tool	Influencing factors	Quality (score)
Naess et al. (23)	2005	Norway	Cohort study	232	15–49	136/96	56(0.2414)	MADRS	①③⑥⑦⑧⑬⑰	8
Zhou et al. (24)	2007	China	Cross-sectional	96	26–45	54/42	35(0.3646)	HAMD	③⑦⑧	5
Guo (25)	2010	China	Cross-sectional	58	26–45	-	22(0.3793)	HAMD	①③⑦⑧	5
Tan et al. (26)	2013	China	Cross-sectional	108	18–45	74/34	49(0.4537)	HAMD	①②⑨⑫㉕	5
Li et al. (9)	2014	China	Cross-sectional	107	28–60	69/38	53(0.4953)	SDS	②③④⑥⑦⑧	8
Li et al. (9)	2014	China	Cross-sectional	107	28–60	69/38	23(0.2150)	SDS	②③④⑥⑦⑧	8
Wang et al. (16)	2014	China	Cross-sectional	69	18–45	56/13	18(0.2609)	HAMD	①②	5
Koivunen et al. (17)	2015	Netherlands	Cohort study	130	16–49	67/73	30(0.2308)	HADS	②③⑥⑪⑭⑱	7
Song et al. (18)	2015	China	Cross-sectional	78	28–45	43/35	28(0.3590)	SDS	②⑮	6
Zuo et al. (27)	2015	China	Cross-sectional	80	18–45	50/30	20(0.2500)	SDS	⑥⑨⑫⑯㉑	5
Tanislav et al. (28)	2015	Multiple European countries	Cross-sectional	2007	18–55	1151/856	202(0.1006)	BDI	②③④⑤⑥	8
Wei et al. (29)	2016	China	Case–control	122	22–45	71/51	52(0.4262)	HAMD	①②⑬⑱㉔	5
Amaricai et al. (30)	2016	Romania	Cross-sectional	72	29–59	54/18	48(0.6667)	BDI	⑩	9
Maaijweea et al. (10)	2016	Netherlands	Cohort study	511	18–50	224/287	86(0.1683)	HADS	⑬⑱㉑	9
McCarthy et al. (31)	2016	United States	Cohort study	64	25–54	35/29	30(0.4688)	CES-D	⑤⑫⑰	6
Wang et al. (32)	2018	China	Cross-sectional	130	18–45	100/30	38(0.2923)	BDI	①⑮	7
Ding (19)	2018	China	Cross-sectional	205	18–55	167/38	71(0.3463)	SDS	⑲⑳	6
Zhang et al. (20)	2019	China	Cross-sectional	248	40–60	168/80	93(0.3750)	SDS	⑥㉓	9
Tang et al. (33)	2019	China	Cross-sectional	153	18–45	81/72	71(0.4641)	HAMD	①②⑥㉕	6
Priya et al. (34)	2021	India	Cross-sectional	150	18–45	110/40	71(0.4733)	CES-D	㉒	8
Hannah et al. (21)	2022	Philippines	Cross-sectional	114	18–49	66/48	23(0.2018)	HADS	④⑩⑲	6
Han et al. (35)	2023	China	Case–control	102	18–59	54/48	30(0.2941)	SDS	①㉑㉒㉖㉗	6
Qiu et al. (36)	2023	China	Case–control	329	18–59	216/113	81(0.2462)	SDS	①㉑㉒㉖㉗	8
Chen et al. (37)	2024	China	Cross-sectional	60	18–59	36/24	28(0.4667)	SDS	①⑮㉑㉓	5
Cai et al. (38)	2025	China	Cross-sectional	160	18–35	131/29	37(0.2313)	HAMD	①⑤㉔	5
Modino et al. (22)	2025	Spain	Cohort study	40	18–50	-	20(0.5000)	HAMD	⑨⑩⑱	8
Modino et al. (22)	2025	Spain	Cohort study	41	18–50	24/17	19(0.4634)	HAMD	⑨⑩⑱	8

MADRS, Montgomery-Asberg Depression Rating Scale; SDS, Self-Rating Depression Scale; HAMD, Hamilton Depression Rating Scale; BDI, Beck Depression Inventory; HADS, Hospital Anxiety and Depression Scale; PHQ-9, Patient Health Questionnaire; CES-D, Center for Epidemiologic Studies Depression Scale. ① NIHSS score, ② lesion location, ③ concomitant hypertension, ④ concomitant diabetes, ⑤ concomitant hyperlipidemia, ⑥ gender, ⑦ smoking status, ⑧ alcohol consumption, ⑨ activities of daily living (ADL), ⑩ Functional status, ⑪ Pain-related indicators, ⑫ Medical burden, ⑬ Educational attainment, ⑭ Hydrocephalus, ⑮ Length of hospitalization, ⑯ Disease knowledge, ⑰ History of depressive symptoms, ⑱ Occupational background, ⑲ Concurrent anxiety, ⑳ Low platelet count, ㉑ household income, ㉒ multiple lesions, ㉓ health insurance coverage, ㉔ lesion area, ㉕ social support, ㉖ SSS score, ㉗ HHI score.

### 3.2 Meta-analysis results

#### 3.2.1 Prevalence of anxiety and depression in young stroke patients

**Figure 2:**
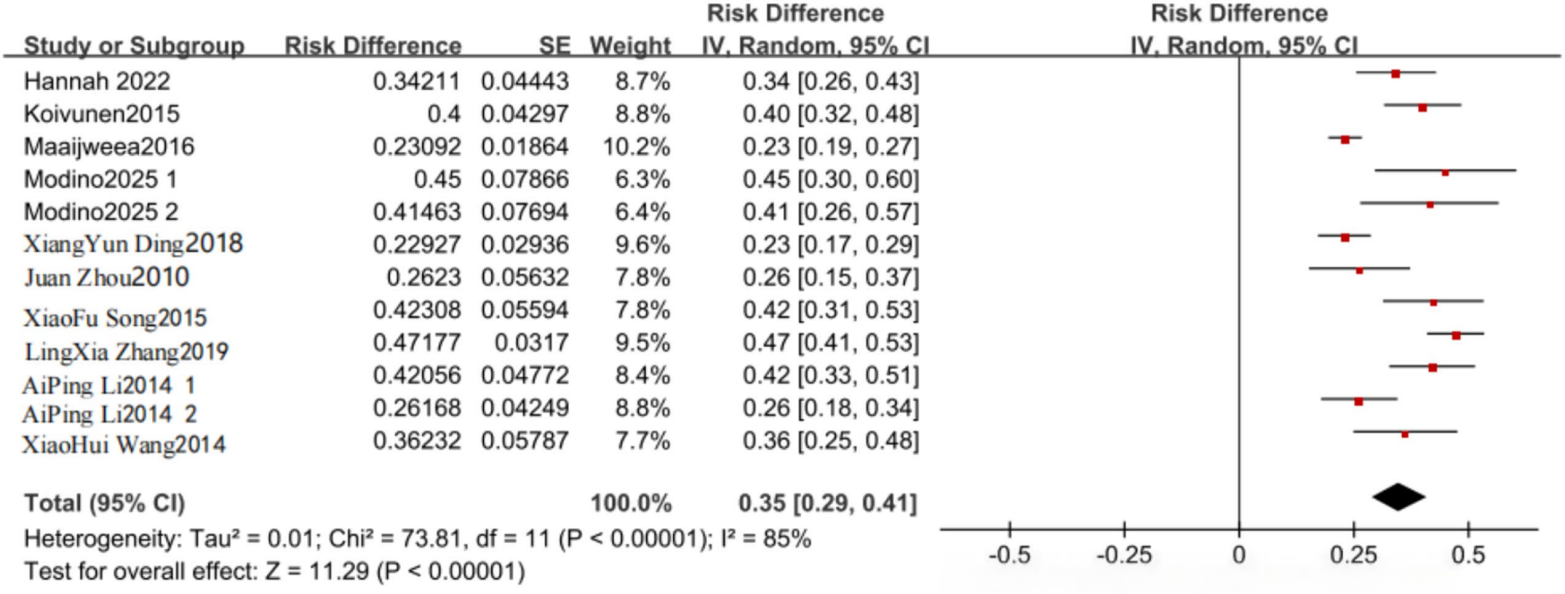
Forest plot of the prevalence of anxiety in young stroke patients.

**Figure 3:**
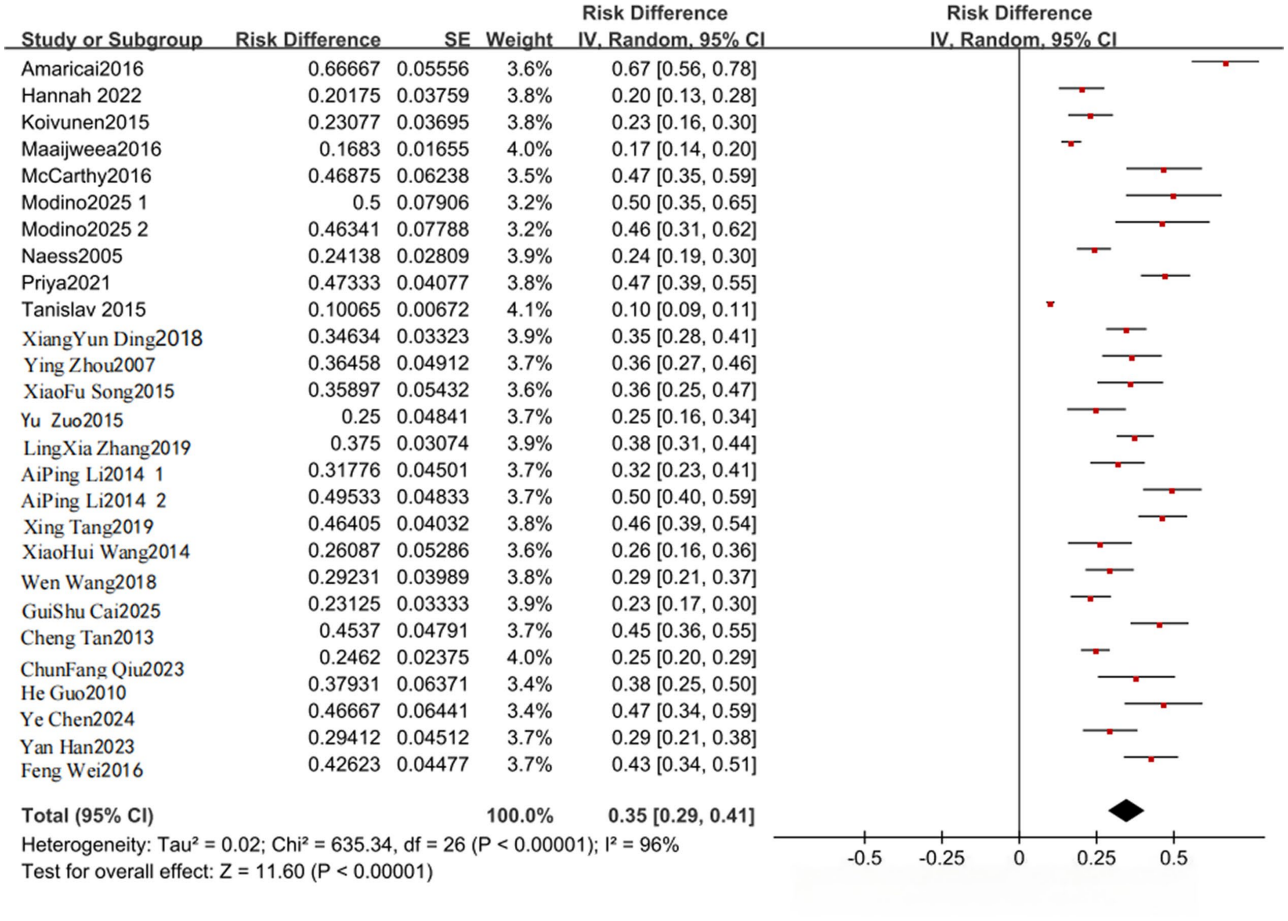
Forest plot of the prevalence of depression in young stroke patients.

Ten studies (9, 10, 15–22) and 25 studies (9, 10, 16–38) reported the prevalence of anxiety and depression, respectively, in young stroke patients. Statistical heterogeneity was present between studies reporting anxiety prevalence (I^2^ = 85%, *p* < 0.001) and depression prevalence (I^2^ = 96%, *p* < 0.001); therefore, random-effects models were applied. The pooled prevalence of anxiety was 35% [95% CI (29–41%)], and the pooled prevalence of depression was also 35% [95% CI (29–41%)] (Figures 2, 3). Although these pooled estimates were numerically identical after rounding, they were derived from different study sets and should be interpreted independently. To further explore heterogeneity, subgroup analyses were conducted according to publication period, country, first-stroke status, and gender. These subgroup analyses were exploratory and were performed to investigate potential sources of heterogeneity rather than to establish prespecified causal contrasts; this point has been clarified to avoid overinterpretation of post-hoc subgroup findings. Specifically, during 2005–2018, the pooled prevalence of anxiety and depression was 32% [95% CI (26–38%)] and 33% [95% CI (26–40%)], respectively, whereas during 2019–2025, the corresponding values were 43% [95% CI (38–47%)] and 32% [95% CI (26–39%)]. In China, the pooled prevalence of anxiety and depression was 35% [95% CI (26–43%)] and 34% [95% CI (30–39%)] compared with 36% [95% CI (26–45%)] and 30% [95% CI (22–38%)] in other countries. Among first-ever stroke patients, the pooled prevalence of anxiety and depression was 32% [95% CI (22–42%)] and 31% [95% CI (24–37%)], whereas among non-first-ever stroke patients it was 37% [95% CI (29–44%)] and 34% [95% CI (26–42%)]. By gender, the pooled prevalence of anxiety and depression was 32% [95% CI (28–36%)] and 36% [95% CI (27–45%)] in male patients and 43% [95% CI (36–49%)] and 37% [95% CI (27–46%)] in female patients (Tables 3, 4).

**Table 3:** Subgroup analysis of the prevalence of anxiety in young stroke patients.

Subgroup	Number of included studies (articles)	Inter-group heterogeneity		Effect model	Meta-analysis results	
		I^2^ value (%)	*p*-value		Proportion	95% CI
Year of publication						
2005–2018	7 (9, 10, 15–19)	80	<0.001	Random	0.32	0.26–0.38
January 2019–September 2025	3 (20–22)	48	0.12	Fixed	0.43	0.38–0.47
Country						
China	6 (9, 15, 16, 18–20)	86	<0.001	Random	0.35	0.26–0.43
Other Countries	4 (10, 17, 21, 22)	84	<0.001	Random	0.36	0.26–0.45
First-ever stroke						
First-ever stroke	3 (9, 10, 17)	87	<0.001	Random	0.32	0.22–0.42
Non-first-ever stroke	7 (15, 16, 18–22)	82	<0.001	Random	0.37	0.29–0.44
Gender						
Male	4 (9, 19–21)	86	<0.001	Random	0.32	0.28–0.36
Female	4 (9, 19–21)	73	<0.001	Random	0.43	0.36–0.49

**Table 4:** Subgroup analysis of the prevalence of depression in young stroke patients.

Subgroup	Number of included studies (articles)	Inter-group heterogeneity		Effect model	Meta-analysis results	
		I^2^ value (%)	*p*		Proportion	95% CI
Publication year						
2005–2018	16 (9, 10, 16–19, 23–32)	96	<0.001	Random	0.33	0.26–0.4
January 2019–September 2025	9 (20–22, 33–38)	86	<0.001	Random	0.32	0.26–0.39
Country						
China	16 (9, 16, 18–20, 24–27, 29, 32, 33, 35–38)	59	<0.001	Random	0.34	0.3–0.39
Other Countries	9 (10, 17, 21–23, 28, 30, 31, 34)	96	<0.001	Random	0.3	0.22–0.38
First-ever stroke						
First-ever stroke	10 (9, 10, 17, 24, 26, 27, 29, 32, 35, 36)	89	<0.001	Random	0.31	0.24–0.37
Non-first-ever stroke	15 (16, 18–23, 25, 28, 30, 31, 33, 34, 37, 38)	96	<0.001	Random	0.34	0.26–0.42
Gender						
Male	17 (9, 19–21, 24–26, 28–30, 32–38)	96	<0.001	Random	0.36	0.27–0.45
Female	17 (9, 19–21, 24–26, 28–30, 32–38)	92	<0.001	Random	0.37	0.27–0.46

#### 3.2.2 Influencing factors of anxiety and depression in young stroke patients

Ten studies (9, 10, 15–22) collectively reported 13 potential factors associated with anxiety in young stroke patients. Meta-analysis showed that alcohol consumption and prior depressive symptoms were consistently associated with anxiety in young stroke patients. Although gender reached statistical significance in the primary analysis, this association became unstable after model switching in sensitivity analysis and should therefore be interpreted cautiously. Twenty-five studies (9, 10, 16–38) reported 27 potential factors associated with depression. Meta-analysis suggested that NIHSS score, alcohol consumption, lesion location, Herth Hope Index (HHI) score, Stroke-related shame (SSS) score, hypertension, diabetes, hyperlipidemia, monthly household income, lesion area, multiple lesions, and length of hospitalization were significantly associated with depression (*p* < 0.05), as shown in Table 5.

**Table 5:** Meta-analysis of influencing factors of depression in young stroke patients.

Influencing factors	Number of included studies (articles)	Heterogeneity test		Meta-analysis results			Sensitivity analysis		
		I^2^(%)	*p*	Effect model	OR(95% CI)	*p*	Effect model	OR(95% CI)	*p*
NIHSS score	11 (16, 23, 25, 26, 29, 32, 33, 35–38)	88	<0.001	Random	3.22[2.04,5.08]	<0.001	Fixed	2.84[2.50,3.22]	<0.001
Alcohol consumption	4 (9, 23–25)	0	0.58	Fixed	3.15[1.85,5.36]	<0.001	Random	3.15[1.85,5.36]	<0.001
Gender	7 (9, 17, 20, 23, 26, 28, 33)	50	0.06	Random	1.27[0.94,1.71]	0.12	Fixed	1.45[1.22,1.71]	<0.001
Smoking status	4 (9, 23–25)	76	0.006	Random	2.27[0.93,5.54]	0.07	Fixed	1.85[1.21,2.83]	0.005
Educational attainment	3 (10, 23, 29)	88	0.0003	Random	1.38[0.40,4.76]	0.61	Fixed	1.43[1.43,2.21]	0.1
Lesion location	5 (9, 16, 26, 29, 33)	10	0.33	Fixed	4.8[2.55,9.06]	<0.001	Random	4.87[2.44,9.70]	<0.001
HHI score	2 (35, 36)	0	0.4	Fixed	1.96[1.42,2.71]	<0.001	Random	1.96[1.42,2.71]	<0.001
SSS score	2 (35, 36)	0	0.47	Fixed	2.04[1.47,2.81]	<0.001	Random	2.04[1.47,2.81]	<0.001
Hypertension	7 (9, 17, 23–26, 28)	30	0.20	Fixed	1.64[1.31,2.04]	<0.001	Random	1.72[1.23,2.39]	<0.001
Diabetes	5 (9, 17, 21, 26, 28)	0	0.61	Fixed	2.15[1.6,2.88]	<0.001	Random	2.15[1.6,2.88]	<0.001
Hyperlipidemia	3 (26, 28, 38)	0	0.49	Fixed	1.53[1.2,1.96]	0.0007	Fixed	1.53[1.2,1.96]	0.0007
Occupational background	3 (10, 17, 29)	84	0.002	Random	0.56[0.18,1.76]	0.32	Fixed	0.51[0.32,0.80]	0.004
Monthly household income	3 (35–37)	82	0.004	Random	1.93[1.18,3.15]	0.009	Fixed	1.67[1.37,2.03]	<0.001
Health insurance coverage	2 (20, 37)	92	0.0003	Random	1.28[0.45,3.67]	0.65	Random	1.32[0.99,1.77]	0.06
Lesion area	2 (29, 38)	0	0.97	Fixed	3.25[1.8,5.87]	<0.001	Random	3.25[1.8,5.87]	<0.001
Multiple lesions	3 (34–36)	4	0.31	Fixed	2.31[1.51,3.55]	<0.001	Random	2.32[1.5,3.6]	0.0002
Length of hospital stay	2 (32, 37)	0	0.96	Fixed	1.62[1.16,2.27]	0.004	Random	1.62[1.16,2.27]	0.004

### 3.3 Sensitivity analysis

#### 3.3.1 Factors influencing anxiety in young stroke patients

Sensitivity analysis showed that, among factors associated with anxiety, the pooled effects for NIHSS score and gender changed after switching statistical models, suggesting that these estimates were unstable and should be interpreted cautiously. By contrast, the pooled estimates for alcohol consumption and prior depressive symptoms remained materially unchanged, supporting the robustness of those findings (Table 6).

**Table 6:** Meta-analysis of influencing factors of anxiety in young stroke patients.

Influencing factors	Number of included studies (articles)	Heterogeneity Test		Meta-analysis results			Sensitivity analysis		
		I^2^(%)	*p*	Effect model	OR(95% CI)	*p*	Effect model	OR(95% CI)	*p*
NIHSS score	2 (15, 16)	99	<0.001	Random	2.37[0.56,10.15]	0.24	Fixed	1.51[1.38,1.65]	<0.01
Gender	2 (9, 20)	36	0.21	Fixed	1.41[1.11,1.81]	0.006	Random	1.53[0.97,2.41]	0.06
Alcohol consumption	2 (9, 10)	0	0.83	Fixed	3.65[1.59,8.42]	0.002	Random	3.62[1.59,8.42]	0.002
History of depressive symptoms	2 (10, 19)	0	0.61	Fixed	4.9[2.95,8.13]	<0.001	Random	4.9[2.95,8.13]	<0.001

#### 3.3.2 Factors influencing depression in young stroke patients

Sensitivity analysis for depression showed that the pooled estimates for gender, smoking status, and occupational background changed after model switching, indicating instability in these associations. Therefore, these factors were not emphasized as robust conclusions in the final interpretation. After omitting individual studies contributing substantial heterogeneity, the associations for gender, smoking, occupational background, and monthly household income became more stable; however, these findings should still be considered exploratory because they were based on post-hoc sensitivity analyses rather than consistent primary models across all studies (Table 5).

### 3.4 Publication bias

Funnel plots for anxiety and depression showed some asymmetry, suggesting possible small-study effects. Egger’s test indicated no clear evidence of publication bias for anxiety studies (*t* = 1.873, *p* = 0.091), but possible publication bias for depression studies (*t* = 2.156, *p* = 0.041). Among factor analyses with at least 10 studies, NIHSS score showed possible publication bias (t = 2.182, *p* = 0.029), whereas other factors did not. Trim-and-fill analysis did not materially change the pooled effect estimates, suggesting that the impact of publication bias on the main conclusions was limited.

## 4 Discussion

This meta-analysis synthesized the available evidence on anxiety and depression among young stroke patients and yielded three main findings. First, both anxiety and depression were highly prevalent in this population, with pooled prevalence estimates of 35%. Second, substantial between-study heterogeneity was observed for both outcomes. Third, alcohol consumption emerged as a common associated factor for both anxiety and depression. For anxiety, prior depressive symptoms showed a relatively robust association, whereas the association with gender was unstable in sensitivity analysis and should be considered exploratory. Taken together, these findings suggest that psychological distress is not a peripheral issue in young stroke survivors, but rather an important component of post-stroke care that should be considered alongside neurological and functional recovery (39).

The relatively high burden observed in this review is clinically meaningful. A recent systematic review focusing specifically on young adults with stroke reported pooled prevalence estimates of 39% for post-stroke anxiety symptoms and 31% for post-stroke depression symptoms, which are broadly comparable with the present findings despite differences in age definitions and included studies (40). In contrast, meta-analyses in the general stroke population have reported somewhat lower pooled estimates, particularly when interview-based methods were used; for example, post-stroke anxiety was estimated at 18.7% by clinical interview and 24.2% by rating scales (41), while post-stroke depression was estimated at 27% overall, including 24% by clinical interview and 29% by rating scales (7). These comparisons suggest that the burden of anxiety and depression in young stroke survivors is at least not lower than that in the broader stroke population. This issue is particularly important because stroke in young adults often occurs during a life stage characterized by work responsibilities, parenting demands, and long-term concerns regarding role resumption and recurrence. Previous studies have further shown that psychological factors are major determinants of quality of life after young stroke, and that depression, recurrent stroke, functional disability, and incomplete return to work are associated with poorer long-term health-related quality of life (42, 43), while more recent evidence has highlighted substantial unmet psychosocial, occupational, and quality-of-life needs in this population (44). Therefore, anxiety and depression in young stroke survivors should be regarded not only as psychiatric comorbidities, but also as clinically important barriers to rehabilitation, social reintegration, and long-term recovery.

The high heterogeneity observed in this review should be interpreted carefully. Several factors likely contributed to this variability. First, the included studies used heterogeneous definitions of “young stroke,” with age ranges spanning approximately 15–60 years in the available evidence base. Second, study characteristics varied considerably with respect to country, study design, stroke subtype, and timing of psychological assessment after stroke. Third, multiple assessment instruments were used, including HADS, HAMA, SAS, SDS, BDI, CES-D, and other scales, and these tools differ in construct coverage, diagnostic thresholds, and sensitivity to somatic versus affective symptoms. This issue is not trivial: in the broader stroke literature, pooled prevalence estimates differ according to whether anxiety or depression is identified by clinical interview or rating scales, and a recent systematic review further suggested that some depression instruments, such as PHQ-9 and HDRS/HAMD, have better diagnostic performance than others for post-stroke depression (7, 41, 45). Accordingly, the pooled prevalence estimates reported here should be interpreted as summary indicators across diverse measurement strategies rather than as fixed epidemiologic constants. The identical rounded prevalence values for anxiety and depression should also be interpreted cautiously, because they reflect pooled estimates derived from different study sets rather than true equivalence between the two conditions.

The subgroup findings should likewise be interpreted as exploratory rather than definitive. Anxiety prevalence appeared higher in women and in patients with non-first-ever stroke, while no statistically meaningful difference was observed between the Chinese and non-Chinese populations in the available subgroup analyses. However, these subgroup contrasts were based on relatively small numbers of studies in some strata and were not designed to establish causal differences. Moreover, 17 of the 26 included studies were conducted in China, which may limit external generalizability. Differences in healthcare systems, rehabilitation pathways, cultural attitudes toward emotional disclosure, and the use of screening tools may all influence the observed prevalence estimates (9, 23, 24). Therefore, the subgroup findings are useful for hypothesis generation but should not be overinterpreted.

With regard to associated factors, alcohol consumption was the only factor linked to both anxiety and depression in the present review. This finding is clinically relevant because alcohol use may reflect a broader cluster of vulnerability, including unhealthy lifestyle behaviors, poorer vascular risk-factor control, and increased psychosocial stress (46). For anxiety specifically, prior depressive symptoms showed a stable association across sensitivity analyses, which is in line with the broader literature demonstrating a close relationship between post-stroke anxiety and depressive symptoms (47). A previous study reported a strong association between post-stroke anxiety and post-stroke depression, and a cohort study further showed that younger age and greater depressive symptoms predicted a higher risk of generalized anxiety after stroke or transient ischemic attack (48). By contrast, the association between female sex and anxiety in the present review became unstable in sensitivity analysis. This suggests that gender may be a potential marker of vulnerability, but the current evidence is insufficient to support a firm conclusion, and the finding should be verified in larger prospective studies. For depression, the relevant factors identified in this study indicate that its development is influenced by multiple factors. This interpretation is consistent with studies showing that depression and employment status are major determinants of long-term quality of life after young stroke, and that stigma and hope are modifiable targets in young and middle-aged stroke populations (43, 49). From a clinical standpoint, these findings support a more integrated model of care in which young survivors with severe deficits, recurrent or extensive lesions, multiple comorbidities, prolonged hospitalization, higher stigma, or poorer psychosocial resources receive earlier psychological screening and more structured psychosocial support (50).

Several limitations should be acknowledged. First, the heterogeneity of the included studies was high, and although exploratory subgroup analyses were conducted, substantial residual heterogeneity remained. Second, the included studies used different instruments, cut-off values, and assessment time points, which may have influenced prevalence estimates and reduced comparability across studies. Moreover, several potentially important sources of heterogeneity, including assessment instrument, study design, stroke subtype, and timing of psychological assessment, could not be formally examined in additional subgroup analyses because these variables were inconsistently reported, some strata contained only a small number of studies, and thresholds across instruments were not sufficiently comparable. Accordingly, further subgroup pooling was considered likely to yield unstable or potentially misleading estimates. Third, there is no uniform definition of the age range for “young stroke patients” across studies, and the available data did not support reliable age-stratified synthesis. This inconsistency likely increased clinical heterogeneity and limited the interpretability of the pooled estimates. Fourth, most included studies were conducted in China, and only Chinese- and English-language publications were included, raising the possibility of regional overrepresentation and language bias. Fifth, some associated factors were supported by only two or three studies, and several estimates were unstable in sensitivity analyses, indicating that these findings should be considered exploratory rather than definitive. Finally, Egger’s test suggested possible publication bias for depression outcomes, although trim-and-fill analysis did not materially alter the pooled effect estimates. Future studies should therefore adopt more standardized age definitions, use more comparable psychological assessment methods, report key methodological and clinical variables more consistently, and include multinational prospective cohorts to better characterize the mental health trajectory of young stroke survivors.

## 5 Conclusion

This review indicates that anxiety and depression are both common among young stroke patients and deserve greater attention in routine stroke care. The observed associations with alcohol consumption, prior depressive symptoms, neurological severity, lesion-related variables, vascular comorbidity, and psychosocial factors suggest that emotional outcomes after young stroke are shaped by both biological and social processes. More standardized and longitudinal research is needed, but the current evidence already supports earlier and more systematic psychological assessment in this population.

## Data Availability

All data extracted and analyzed during this meta-analysis are included in this published article, further inquiries can be directed to the corresponding author.
